# Digital Light Processing of Zirconia Suspensions Containing Photocurable Monomer/Camphor Vehicle for Dental Applications

**DOI:** 10.3390/ma16010402

**Published:** 2023-01-01

**Authors:** Seo-Young Yang, Young-Hag Koh, Hyoun-Ee Kim

**Affiliations:** 1 Interdisciplinary Program in Precision Public Health, Korea University, Seoul 02841, Republic of Korea; 2 School of Biomedical Engineering, Korea University, Seoul 02841, Republic of Korea; 3 Department of Materials Science and Engineering, Seoul National University, Seoul 08826, Republic of Korea

**Keywords:** 3D printing, photopolymerization, digital light processing, zirconia, dental crowns

## Abstract

This study reports the utility of solid camphor as a novel diluent in photocurable hexanediol diacrylate (HDDA) monomer to manufacture 4 mol% yttria partially stabilized zirconia (4Y-PSZ) components for dental applications by digital light processing (DLP). The use of a 65 wt% HDDA–35 wt% camphor solution allowed 4Y-PSZ suspensions to have reasonably low viscosities (1399 ± 55.8 mPa·s at a shear rate of 75 s^−1^), measured by a cone/plate viscometer, at a high solid loading of 48 vol%, where 4Y-PSZ particles prepared by calcination of as-received 4Y-PSZ granules, followed by a ball-milling process, were used with assistance of a dispersant. These 4Y-PSZ suspensions could be successfully applied to our custom-made DLP machine for manufacturing 4Y-PSZ components. To this end, several processing parameters, including layer thickness of 4Y-PSZ suspension, UV illumination time for layer-by-layer photocuring process, and initial dimensions of 4Y-PSZ objects, were tightly controlled. As sintering temperature increased from 1300 °C to 1500 °C, relative density and grain size of 4Y-PSZ objects increased, and cubic phase content also increased. Thus, after sintering at the highest temperature of 1500 °C for 3 h, high mechanical properties (biaxial flexural strength = 911 ± 40.7 MPa, hardness = 1371 ± 14.4 H_v_) and reasonably high optical transmittance (translucency parameter = 7.77 ± 0.32, contrast ratio = 0.809 ± 0.007), evaluated by a spectrophotometer, were obtained due to a high relative density (97.2 ± 1.38%), which would be useful for dental applications.

## 1. Introduction

Yttria (Y_2_O_3_)-doped zirconia (ZrO_2_) ceramics are one the most widespread materials for manufacturing dental crowns because they can offer good esthetics with shades and colors resembling those of natural teeth and excellent long-term mechanical functions [1,2,3]. In particular, 3 mol% yttria stabilized tetragonal zirconia polycrystal (3Y-TZP) has been clinically used for a long period of time owing to its unusually high fracture strength and fracture toughness [4]. More specifically, when stressed, grains with a metastable tetragonal (T) phase can be transformed into grains with a more stable monoclinic (M) phase. This stress-induced phase transformation can cause considerable volume expansion and induce residual compressive stress, thus hindering crack propagation at a crack tip [1,4]. However, 3Y-TZP lacks translucency, limiting its wider clinical uses. Thus, attention has been shifted to 4 mol% and 5 mol% yttria partially stabilized zirconia (known as 4Y-PSZ and 5Y-PSZ, respectively) as they can provide significantly enhanced translucency at a cost of mechanical properties [1,4,5,6,7]. This is attributed to an increase in the fraction of optically isotropic cubic (C) phase coexisting with the tetragonal (T) phase [7].

From the viewpoint of manufacturing dental zirconia components (e.g., dental crowns, abutments, and implants), 3D printing techniques have recently attracted increasing attention as an alternative to traditional dental computer-aided design and computer-aided manufacturing (CAD/CAM) technology [8,9,10]. In particular, photopolymerization–based 3D printing techniques such as digital light processing (DLP) and stereolithography (SLA) can construct zirconia components by photopolymerizing thin layers of a zirconia suspension in a layer-by-layer fashion according to predetermined 3D designs. Thus, a high degree of design freedom with a high accuracy can be achieved, which is crucial for manufacturing dental zirconia ceramics with external and internal shapes tailored for individual patients [8,9,11,12]. In addition, such an additive manufacturing approach can avoid high consumption of cutting tools and great losses of discarded materials caused by dental CAD/CAM.

However, to manufacture dental zirconia components, special care should be taken to optimize processing parameters for the photopolymerization-based 3D printing process since flaws and residual pores after sintering can severely deteriorate translucency [13,14] and mechanical properties [15]. One of the most crucial steps is to prepare zirconia suspensions with high solid loadings, where fine zirconia particles should be uniformly dispersed in photopolymerizable vehicles, and thus high densification can be achieved after sintering [10,16,17,18,19,20]. In addition, they should have reasonably low viscosities and good flowability to make thin layers of zirconia suspensions suitable for a layer-by-layer process [21,22]. However, a trade-off relationship between solid loading and viscosity makes it troublesome to achieve reasonably low viscosities at high solid loadings [12,18].

Thus, considerable efforts have been made to reduce the viscosities of zirconia suspensions, for example, by utilizing dispersants [23] and surface modified zirconia particles [24]. In addition, multifunctional monomers used as major photopolymerizable vehicles to offer strength after 3D printing can be blended with diluents. More specifically, low-viscosity monofunctional monomers as reactive diluents can be effective in reducing viscosities [25,26]. However, they can also be photopolymerized by 3D printing, often making the debinding process more troublesome. On the other hand, organic solvents with extremely low viscosities (e.g., decalin [27], octanol [28], methanol [26], and water [29]) can effectively reduce viscosities of multifunctional monomers. In addition, these nonreactive diluents can remain in the photopolymerized phase and then removed by evaporation, thus facilitating the debinding process. However, inhomogeneous shrinkage of 3D printed objects caused by drying of organic solvents has potential risks of cracking and distortion.

Recently, our group has proposed that solid camphor (C_10_H_16_O) [30,31]) could be dissolved in photocurable monomers, thus offering significantly reduced viscosities of ceramic suspensions. In addition, camphor dissolved in photocurable monomers can recrystallize after photopolymerization and camphor crystals are removed via sublimation in a solid state [30,32], thus eliminating drying shrinkage, unlike liquid organic diluents. Thus, the main purpose of the present study was to utilize the above-mentioned solid camphor as a diluent to formulate 4Y-PSZ suspensions with reasonably low viscosities at high solid loadings, which could be used to manufacture high-quality 4Y-PSZ for dental applications using the DLP process. To this end, we carefully controlled size distribution of 4Y-PSZ particles by calcination of as-received 4Y-PSZ granules followed by a ball-milling process and adjustment of dispersant content to uniformly disperse 4Y-PSZ particles with low viscosities. The effect of camphor addition into photocurable hexanediol diacrylate (HDDA) monomer on the reduction in viscosities of 4Y-PSZ suspensions was examined first. After which, a maximum solid loading with reasonably low viscosity available for DLP process was determined. In addition, several processing parameters, including layer thickness of 4Y-PSZ suspensions, UV illumination time for layer-by-layer photocuring process, and initial dimensions of 4Y-PSZ objects, were tightly controlled. Green 4Y-PSZ components were then manufactured using our custom-built DLP machine followed by heat-treatment for debinding to remove organic phases (HDDA, camphor, dispersant, and photoinitiator) and sintering to densify 4Y-PSZ. In particular, 4Y-PSZ components were sintered at various temperatures (1300 °C, 1400 °C, and 1500 °C) for 3 h, in order to examine changes in densification behaviors, grain size evolution, mechanical properties (biaxial flexural strength and Vickers hardness), and optical properties.

## 2. Materials and Methods

### 2.1. Starting Materials

Components of zirconia suspensions prepared using an HDDA–camphor solution as a liquidus vehicle for DLP process are summarized in Table 1. In particular, 4 mol% yttria-partially stabilized zirconia (4Y-PSZ) powder supplied by Tosoh Co. (Tokyo, Japan). was used to obtain high translucency. As-received spherical granules with diameters of several tens of microns composed of nanoparticles with a small amount of binder were calcined at 1000 °C for 1 h in air to remove binders. Calcined granules were then crushed into smaller particles by ball-milling for 24 h at a rotation speed of 90 rpm. Shapes of the as-received granules and ball-milled powders were then characterized by field emission scanning electron microscopy (FE-SEM: JSM-6701F, JEOL Techniques, Tokyo, Japan). Prior to FE-SEM observations, the surfaces of the samples were coated with Au films using a sputter-coater (Cressington 108 auto, Cressington, Watford, UK) for 180 s at a current of 10 mA under 0.09 mbar. High-resolution FE-SEM images were then taken in secondary electron imaging (SEI) mode, where an accelerating voltage of 10 kV and an emission current of 10 μA were employed, while a working distance was in the range of 8–11 mm depending on magnifications. Their size distributions were characterized by a laser particle size analyzer (CILAS 1090, Orleans, France). All other reagents were used as purchased without further purification and/or treatment.

### 2.2. Zirconia Suspensions Preparation

To prepare 4Y-PSZ suspensions, an HDDA/camphor solution with a camphor content of 35 wt% was first prepared by dissolving solid camphor in liquid HDDA using a planetary centrifugal mixer (Hantech Co., Ltd., Gyeonggi-do, Republic of Korea) for 5 min at a rotation speed of 1000 rpm, where zirconia (ZrO_2_) balls were used as media for vigorous mixing. After which, a predetermined amount of dispersant was then mixed with the prepared HDDA/camphene solution for 10 min. After that, a predetermined amount (40 vol%, 45 vol%, and 48 vol%) of 4Y-PSZ powder was gradually added into the dispersant-containing HDDA–camphor solution and then vigorously mixed by a planetary centrifugal mixer. In order to effectively reduce viscosities of 4Y-PSZ suspensions, various dispersant contents (2–5 wt% with respect to the 4Y-PSZ content of 40 vol%) were examined. In addition, effects of 4Y-PSZ powder content (40 vol%, 45 vol%, and 48 vol%) on viscosities of 4Y-PSZ suspensions were examined. At the final stage, a predetermined amount (2 wt% with respect to the HDDA content) of photoinitiator was mixed with the 4Y-PSZ suspensions for 10 min.

Rheological behaviors of 4Y-PSZ suspensions prepared with HDDA and HDDA–camphor solution were characterized using a cone/plate viscometer (DV3T-CP, Bookfield, MA, US). Apparent viscosity (*η*) was monitored as a function of the shear rate (d*γ*/dt), ranging from 75 s^−1^ to 150 s^−1^. Three suspensions were tested for each condition to obtain mean and standard deviation. In addition, rheological behaviors of 4Y-PSZ suspensions with different dispersant contents and 4Y-PSZ powder contents were examined. Three suspensions were tested for each condition to obtain mean and standard deviation.

### 2.3. Optimization of DLP Process for 4Y-PSZ

To manufacture 4Y-PSZ crowns, a custom-built DLP printer (Veltz3D, Incheon, Republic of Korea) was utilized, whose basic concept was previously proposed by our group [27]. In this 3D printer, a 4Y-PSZ suspension can be extruded by a step motor and then spread uniformly using a recoater. Thus, highly concentrated ceramic suspensions even with relatively high viscosities can be uniformly deposited without difficulty, unlike conventional DLP process. For the photopolymerization process, a digital micromirror device (DMD) employing a dynamic mask of 1920 × 1080 pixels was used, which could illuminate a UV power of ~14.95 mW/cm^2^ at a peak wavelength of ~405 nm.

To determine the optimum exposure time for complete photopolymerization of 50-micron-thick layers used in this study, a range of UV exposure times (3–15 s) were applied to layers. Their photopolymerized thicknesses were then measured by a micrometer. The degree of line broadening caused by UV scattering by 4Y-PSZ particles was characterized by measuring differences between designed diameters (200–1000 μm) and photopolymerized diameters using an optical microscope (DIMIS-M, Siwon optical technology, Gyeonggi-do, Republic of Korea). Three and six samples were tested for measurements of photopolymerized thicknesses and diameters, respectively, in order to obtain the mean and standard deviation.

### 2.4. Manufacturing of 4Y-PSZ Disks

To optimize processing parameters for our DLP process, 4Y-PSZ components with a relatively simple shape (a disk geometry with a diameter of 14.4 mm and a height of 1.3 mm) were first manufactured. Their microstructures, sintering behaviors, and mechanical and optical properties were characterized. To this end, 50-micron-thick layers of 4Y-PSZ suspensions were photopolymerized selectively according to predetermined 2D designs for 5 s in a layer-by-layer fashion.

**Figure 1:**
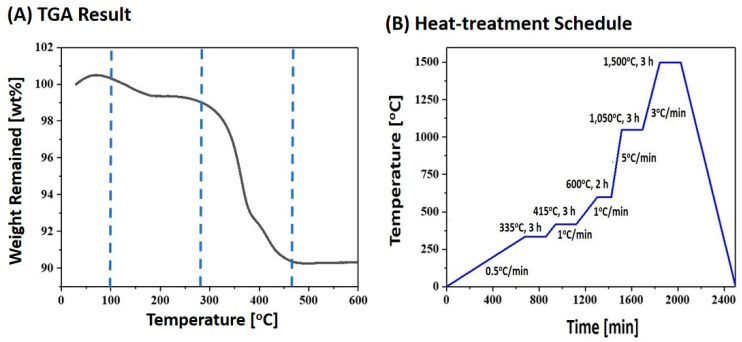
(**A**) TGA result of an as-manufactured 4Y-PSZ component as a function of temperature (**B**) heat-treatment schedule for debinding with a multi-step debinding using slow heating rates below 600 °C to avoid the formation of defects and sintering at 1500 °C for 3 h.

As-manufactured 4Y-PSZ components were then washed with ethanol several times to completely remove uncured suspensions. These 4Y-PSZ components were then heat-treated to remove organic phases (i.e., camphor, photocured HDDA, dispersant, and photoinitiator) via debinding and densify 4Y-PSZ via sintering. To determine the debinding schedule, thermogravimetric analysis (TGA; Mettler-toledo International Inc., Greifenese, Switzerland) was performed to monitor the weight losses of a sample during heating. The sample was heat-treated at a heating rate of 5 °C/min up to 600 °C in nitrogen, and then its weight loss was recorded. Two samples were tested to obtain reliable results. A representative TGA-DTA curve is shown in Figure 1A. Thermal decomposition started above 100 °C due to removal of diluent, dispersant, and photoinitiator. With a further increase in temperature, weight loss increased very rapidly due to intensive removal of photopolymerized HDDA, particularly at the temperature range of ~270–460 °C. Based on this TGA analysis, a multi-step debinding schedule, particularly using slow heating rates below 600 °C, was established (Figure 1B) in order to avoid the formation of defects, such as interfacial delamination between deposited layers and cracking within layers generally associated with rapid thermal decomposition of photopolymerized HDDA. After that, 4Y-PSZ components were sintered at various temperatures (1300 °C, 1400 °C, and 1500 °C) for 3 h to examine effects of sintering temperatures on densification, flexural strength, and optical transmittance.

### 2.5. Characterization of 4Y-PSZ Disks

To examine the utility of 4Y-PSZ suspensions with an HDDA–camphor vehicle for the DLP process, 3D shapes and microstructures of as-manufactured components were characterized by optical microscopy and FE-SEM, respectively. In addition, their dimensions (i.e., diameter and thickness) were measured by a micrometer to evaluate the accuracy of our printing technique.

The 4Y-PSZ components sintered at various temperatures (1300 °C, 1400 °C, and 1500 °C) were characterized in terms of 3D shapes, microstructures, mechanical properties, and optical properties. Dimensions of sintered 4Y-PSZ components were measured to evaluate their sintering shrinkages. Sintering shrinkages were then calculated by dividing diameters of sintered 4Y-PSZ disks by those of as-manufactured disks. Five samples were tested to obtain the mean and standard deviation. To calculate relative densities of sintered components, their densities were measured using Archimedes’ principle and then divided by theoretical density of 4Y-PSZ (6.07 g/cm3 according to manufacturer’s specification). Ten samples were tested to obtain the mean and standard deviation. Microstructures of sintered 4Y-PSZ components were characterized by FE-SEM. Grain sizes of 4Y-PSZ samples sintered at 1300 °C, 1400 °C, and 1500 °C for 3 h were measured by the linear intercept method [33]. Three samples were tested for each condition and five lines were taken from each sample, in order to obtain mean and standard deviation.

Crystalline phases of sintered specimens were examined by X-ray diffraction. An X-ray diffractometer (DMAX-2500, Rigaku Corporation, Tokyo, Japan) with Cu Kα radiation operating at 40 kV and 200 mA was used. Diffraction patterns of specimens were recorded between 2θ ranging from 10° to 80° with a step size of 0.04°. Peaks were indexed by matching their diffraction angles and relative intensities to those of tetragonal (JCPDS card no. 01-079-1767) and cubic (JCPDS card no. 01-081-1550) phases of crystalline zirconia. Contents of tetragonal and cubic phases were calculated by Rietveld refinement using X’Pert Highscore Plus software 3.0 (Malvern Panalytical, Worcestershire, UK).

Mechanical properties of 4Y-PSZ disks sintered at various temperatures (1300 °C, 1400 °C, and 1500 °C) were characterized in terms of strength and hardness for dental applications. For strength evaluations, biaxial flexural test in accordance with ISO 6872 for ceramic materials in dentistry was employed [34,35]. Disk-shaped specimens with a dimeter of ~12.2 mm were ground and then polished to obtain flat and smooth surfaces with a thickness of 1 mm. For biaxial flexural strength tests, a load was applied using a flat punch with a diameter of 1.46 mm to the center of the specimen supported by three balls with a diameter of 5 mm positioned 120° part on a support circle with a diameter of 11.5 mm. A constant crosshead speed of 1 mm/min was applied using a screw-driven load frame (OTU-05D; Oriental TM Corp., Siheung-si, Gyeonggi-do, Republic of Korea) until specimens were fractured. Load (*p*) at fracture was then recorded. Biaxial flexural strength (*σ*) could be computed based on load (*p*) and specimen thickness (*b*) using the following equations [34]:

*σ* = −0.2387 × *p*(X − Y)/*b*^2^ (1)

where

X = (1 + ν)*ln*(*r*_2_/*r*_3_)^2^ + [(1 − ν)/2*ln*(*r*_2_/*r*_3_)^3^] (2)

Y = (1 + ν)*ln*(*r*_1_/*r*_3_)^2^ + [(1 − ν)/2*ln*(*r*_1_/*r*_3_)^3^] (3)

where ν is Poisson’s ratio (assuming 0.25) [35] and *r*_1_, *r*_2_, and *r*_1_ are radii of support circle, loaded area, and disk specimen, respectively. Three specimens were tested for each condition in order to obtain the mean and standard deviation.

For hardness evaluations, Vickers hardness test was performed in accordance with ASTM C1372-2015 [36]. A force (F) of 9.8 N was applied to polished surfaces of specimens for 10 s using a Vickers hardness tester (HV-100; Mitutoyo Co., Kawasaki, Japan). The two axes of the diamond shaped indentation were measured by FE-SEM and then were averaged to obtain the average diagonal length (*d*). Vickers hardness (H_v_) was then calculated using the following equations:

H_v_ = 0.1891 × F/d2 (4)

Five specimens were tested for each condition to obtain mean and standard deviation.

Optical properties of 4Y-PSZ disks sintered at various temperatures (1300 °C, 1400 °C, and 1500 °C) were evaluated by measuring their contrast ratio (CR) and translucency parameter (TP) values using a spectrophotometer (CM-700d, Konica Minolta Inc., Tokyo, Japan) [37,38]. All specimens used for measurements had the same thickness (1 mm). CEILAB coordinates (L *, a *, and b *) were recorded against white and black calibrated background boards. L* indicates the lightness of the color ranging from 0 to 100, where L * = 0 and L * = 100 indicate black and white, respectively (higher numbers being brighter) [39], and a * and b * represent changes in saturation from red to green and from blue to yellow, respectively. To quantitatively evaluate the translucency of each sintered specimen, the contrast ratio (CR) was calculated by considering white reference backing (Y_W_) and black backing (Y_B_), as follows [40]:

CR = Y_B_/Y_W_ (5)

CR values of 0.0 and 1.0 values correspond to transparent and totally opaque materials, respectively. TP was also calculated based on CEILAB coordinates, as follows:

TP = [(L *_B_ − L *_W_)^2^ + (a *_B_ − a *_W_)^2^ + (b *_B_ − b *_W_)^2^]^1/2^ (6)

where the subscripts B and W represent color coordinates over black and white backgrounds, respectively [41].

### 2.6. Statistical Analysis

All data were expressed as the mean ± standard deviation. The statistical analysis was performed by a one-way analysis of variance (ANOVA) with MATLAB (The MathWorks, Inc., Natick, MA, USA) with Tukey–Kramer Post Hoc Test (OrginLab Corp., Northampton, MA, USA). *p* values less than 0.05 were considered statistically significant.

## 3. Results and Discussion

### 3.1. Preparation of 4Y-PSZ Powders for DLP Process

**Figure 2:**
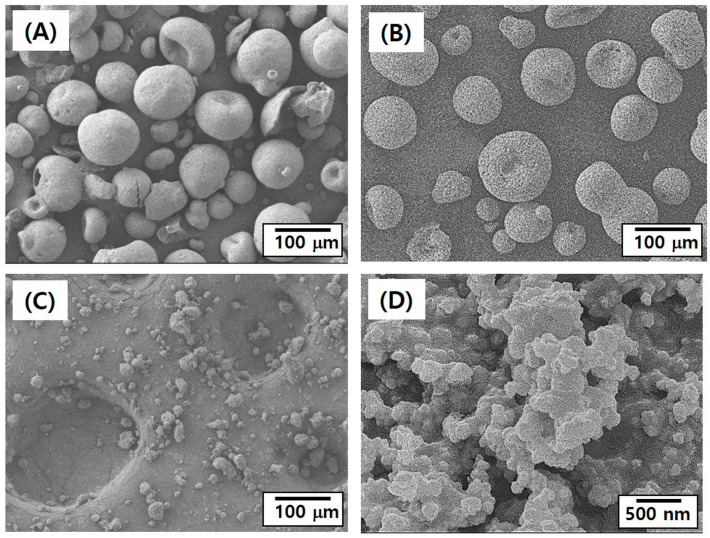
Representative FE-SEM images of (**A**) as-received 4Y-PSZ granules, (**B**) calcined granules, and ball-milled powders at low (**C**) and high (**D**) magnifications.

To utilize our DLP technique for manufacturing dental 4Y-PSZ components with high quality, it is crucial to prepare 4Y-PSZ suspensions with desired rheological properties for the 3D printing process and excellent densification behavior during sintering at high temperatures. Fundamentally, these properties are strongly influenced by characteristics of powders, including their size, specific surface area, and shapes [11,27]. For example, 4Y-PSZ granules supplied by Tosoh co. had very large sizes at several tens of micron (Figure 2A), making it difficult to obtain high solid loading in 4Y-PSZ suspensions required for high densification, mechanical properties, and optical translucency. In addition, they had a small amount (~4 wt%) of polymeric binder, which was used to obtain a spherical shape by binding 4Y-PSZ nanoparticles. Thus, as-received 4Y-PSZ granules were calcined at 1000 °C for 3 h to remove polymeric binder and induce partial necking between nanoparticles. There is no notable change in shape or sizes (Figure 2B). These calcined porous granules could be effectively crushed into finer powders by ball-milling (Figure 2C). Particles with very different sizes were obtained, which could reduce viscosities of 4Y-PSZ suspensions compared to particles with a narrow particle size distribution [11,12]. In addition, 4Y-PSZ nanoparticles were partially bonded together due to a relatively high calcination temperature of 1000 °C (Figure 2D).

**Figure 3:**
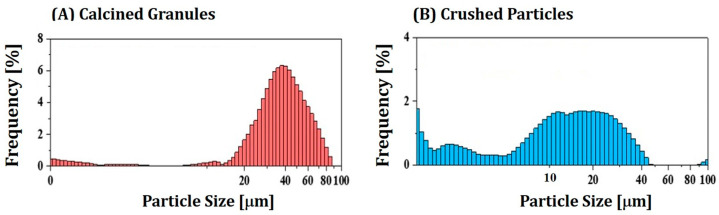
Particle size distributions of (**A**) calcined 4Y-PSZ granules and (**B**) ball-milled 4Y-PSZ powders.

Calcined granules showed relatively large sizes in the range of 10–80 mm (Figure 3A). A mean size was 38.36 mm. Although these large granules could be partially crushed during mixing with a photocurable HDDA–camphor solution by high-power planetary centrifugal mixing, they were not mixed favorably due to their large sizes when solid loadings were higher than 40 vol%. On the other hand, ball-milled powders showed a broad size distribution, containing both large particles (10–40 mm in size) and small particles (<10 μm) (Figure 3B). These micron-sized particles with a wide distribution could be effectively used for preparing 4Y-PSZ suspensions with reasonable viscosities at high solid loadings, since smaller particles can fill voids formed between large particles [12]. However, when nano-sized 4Y PSZ particles were used, it was difficult to increase solid loading presumably due to their large surface areas.

### 3.2. Effect of Camphor Addition on Viscosity of 4Y-PSZ Suspensions

**Figure 4:**
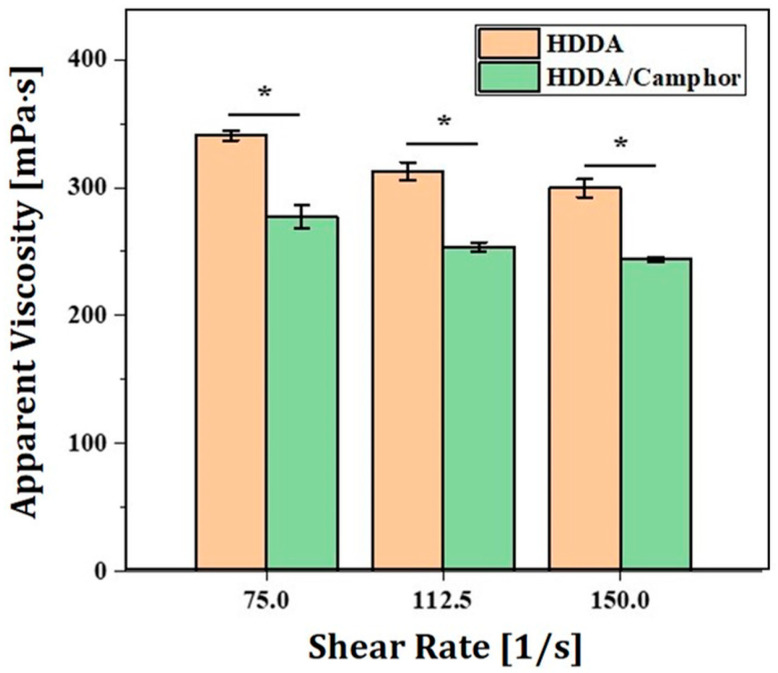
Apparent viscosities at various shear rates observed for 4Y-PSZ suspensions prepared using HDDA and HDDA/camphor vehicle (solid loading = 40 vol% and dispersant content = 4 wt%) (* *p* < 0.05).

To achieve excellent 3D printing capability and high densification for desired mechanical and optical properties, 4Y-PSZ suspensions should have reasonably low viscosities at high solid loadings. Thus, camphor was used as a diluent. The basic concept behind this has been reported by our group previously, in order to reduce viscosities of 4Y-PSZ suspensions with high solid loadings [30]. The effect of camphor addition on viscosities of 4Y-PSZ suspension was examined. Two types of 4Y-PSZ suspensions with a solid loading containing 4 wt% of the dispersant were prepared using HDDA and HDDA/camphor vehicles. Figure 4 shows their rheological behaviors characterized by a cone/plate viscometer. Both suspensions showed that viscosities decreased with an increase in shear rate ranging from 75 s^−1^ to 150 s^−1^, indicating shear thinning behaviors [12]. However, 4Y-PSZ suspensions prepared using an HDDA/camphor vehicle showed lower viscosities over given shear rates than those prepared using HDDA vehicle. It is reasonable to suppose that this trend—effect of camphor as a diluent—would be more pronounced with an increase in solid loading.

### 3.3. Effect of Dispersant Content on Viscosities of 4Y-PSZ Suspensions 

**Figure 5:**
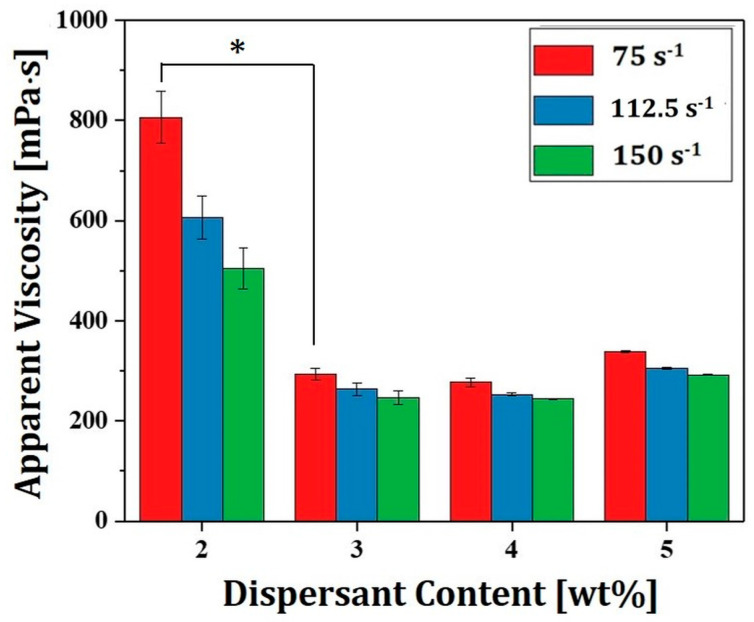
Apparent viscosities at various shear rates observed for 4Y-PSZ suspensions prepared using different dispersant contents (2 wt%, 3 wt%, 4 wt%, and 5 wt%) (* *p* < 0.05).

Effect of dispersant content on viscosities of 4Y-PSZ suspension prepared using a HDDA/camphor vehicle was also examined. Results are shown in Figure 5. To induce effective repulsion between 4Y-PSZ particles, DISPERBYK-2001, a solution of a structured acrylate copolymer with pigment-affinic groups, was employed as the dispersant due to its high solubility in HDDA. As the dispersant content increased from 2 wt% to 3 wt%, the viscosity decreased remarkably from 806 ± 52 mPa·s to 293 ± 12 mPa·s at a shear rate of 75 s^−1^. In addition, a higher dispersant content of 4 wt% resulted in a further reduction in viscosity to a certain degree. However, the viscosity increased at a high dispersant content of 5 wt% due to a thick layer of the dispersant. On the basis of this test, a dispersant content of 4 wt% was employed to prepare 4Y-PSZ suspensions with higher solid loadings (e.g., 45 vol% and 48 vol%).

### 3.4. Effect of Solid Loading on Viscosities of 4Y-PSZ Suspensions

**Figure 6:**
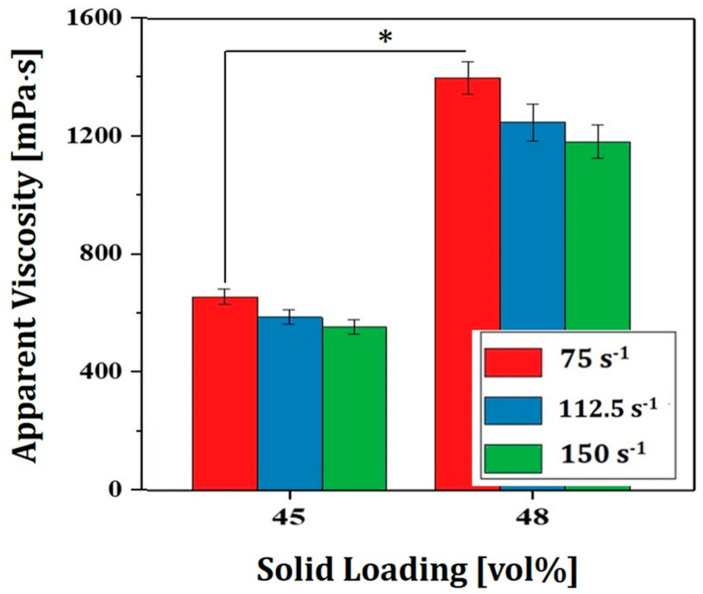
Apparent viscosities at various shear rates observed for 4Y-PSZ suspensions prepared using various solid loadings (45 vol% and 48 vol%) (* *p* < 0.05).

To obtain high densification after sintering required for desired mechanical and optical properties, 4Y-PSZ suspensions should have reasonably high solid loadings, while their viscosities should be sufficiently low for a 3D printing process. To this end, the effect of solid loading (45 vol% and 48 vol%) on viscosities of 4Y-PSZ suspensions was examined. Results are shown in Figure 6. Both suspensions showed a decrease in viscosity with an increase in shear rate, indicating that their shear thinning behaviors were similar to those prepared at a lower solid loading of 40 vol%. However, their viscosity increased notably with an increase in solid loading. For example, at a shear rate of 75 s^−1^, viscosities of 657 ± 26.9 mPa∙s and 1399 ± 55.8 mPa∙s were observed at solid loadings of 45 vol% and 48 vol%, respectively.

Although a range of viscosities could be successfully utilized to manufacture zirconia components by various DLP machines (Table 2), a lower viscosity is preferred to make uniform layers for layer-by-layer photocuring processes and avoid delamination between photocured layers. However, it should be noted that viscosities should depend on shear rates. In this respect, our HDDA–camphor vehicle is expected to offer a new strategy to formulate highly concentrated zirconia suspensions with reasonably low viscosities. In addition, camphor crystals formed in green 4Y-PSZ objects after photocuring can be removed by sublimation in the solid state, thus avoiding defects caused by drying unlike liquid organic diluents and facilitating the debinding process by leaving pores in green 4Y-PSZ objects.

### 3.5. Photopolymerization Behavior of 4Y-PSZ Suspensions with Camphor/HDDA Vehicle

**Figure 7:**
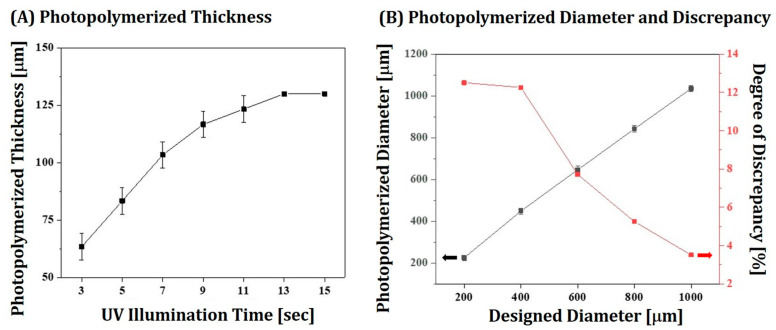
(**A**) Thicknesses of photopolymerized layers for various UV illumination times (3–15 s) and (**B**) diameters of photopolymerized disks according to designed diameters and discrepancy in relation to designed diameters.

To optimize processing parameters for our DLP process, cure depth and width of a 4Y-PSZ suspension prepared with a HDDA/camphor vehicle were examined. Layers of a 4Y-PSZ suspension were photopolymerized for various time periods and their cured thicknesses measured using a micrometer. Results are displayed in Figure 7A. As the UV illumination time increased from 3 s to 11 s, the cure depth increased from 63.3 ± 5.8 μm to 116.7 ± 5.8 μm. This finding suggests that camphor dissolved in HDDA can negligibly deteriorate the photopolymerization capability of 4Y-PSZ suspensions. On the basis of this evaluation, a photopolymerization time of 5 s was employed to manufacture 4Y-PSZ components, which could completely photopolymerize 50-μm-thick layers and offer good bonding between deposited layers.

To closely replicate complicated structures of dental components required for individual patients, special care should be taken to design initial dimensions, since 4Y-PSZ particles with higher refractive index than HDDA could scatter incident UV light during photopolymerization, leading to considerably expanded dimensions [42,43]. Thus, the discrepancy between designed and photopolymerized disks was examined by measuring their diameters. Results are displayed in Figure 7B. When the initial diameters were relatively small, notably enlarged diameters were obtained indicated by the black arrow (225 ± 11.9 μm and 449 ± 14.1 μm for 200 μm and 400 μm, respectively). However, the discrepancy (red arrow) decreased with an increase in the initial diameter. On the basis of this evaluation, the initial dimensions of dental crowns could be precisely designed for individual cases.

### 3.6. Microstructures of As-Manufactured 4Y-PSZ Disks 

**Figure 8:**
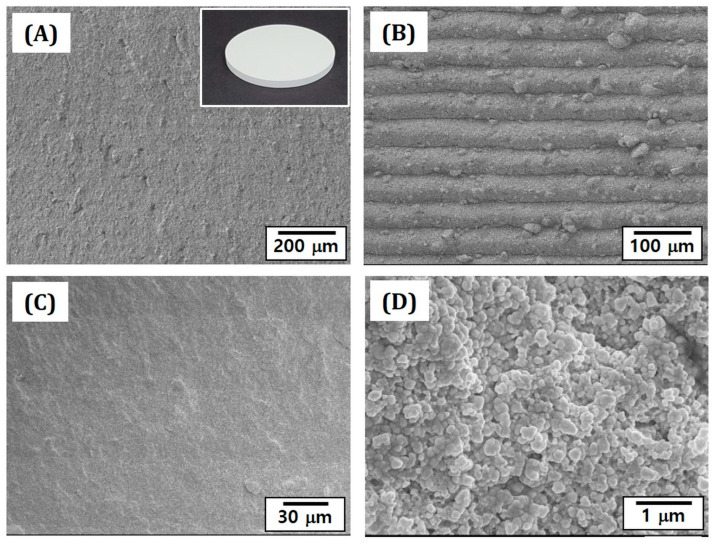
Representative FE-SEM images showing free surfaces (**A**,**B**) and fracture surfaces (**C**,**D**) of as-manufactured 4Y-PSZ disk in the building direction. The inset in Figure 8 (**A**) shows representative optical image of the as-manufactured 4Y-PSZ disk (diameter = 14.4 mm).

Disk-shaped 4Y-PSZ specimens were manufactured by our photopolymerization-based 3D printing using a 4Y-PSZ suspension containing an HDDA/camphor vehicle with a solid loading of 48 vol%. The inset in Figure 8A shows a representative optical image of an as-manufactured 4Y-PSZ disk. The free surface of the disk showed no interfacial delamination between layers (Figure 8A). This finding suggests that 50-μm-thick layers of a 4Y-PSZ suspension could be completely photopolymerized by UV illumination for 5 s. However, grooved layers were visible in the 3D printing direction at a higher magnification (Figure 8B), which is often observed in the DLP process due to UV scattering by ceramic particles with higher refractive indices than monomers [27]. On the other hand, the fracture surface of the disk showed a relatively dense microstructure without large voids or interfaces between layers (Figure 8C). In addition, fine 4Y-PSZ particles were uniformly dispersed throughout the disk (Figure 8D).

### 3.7. Effect of Sintering Temperature on Densification Behavior of 4Y-PSZ Disks

**Figure 9:**
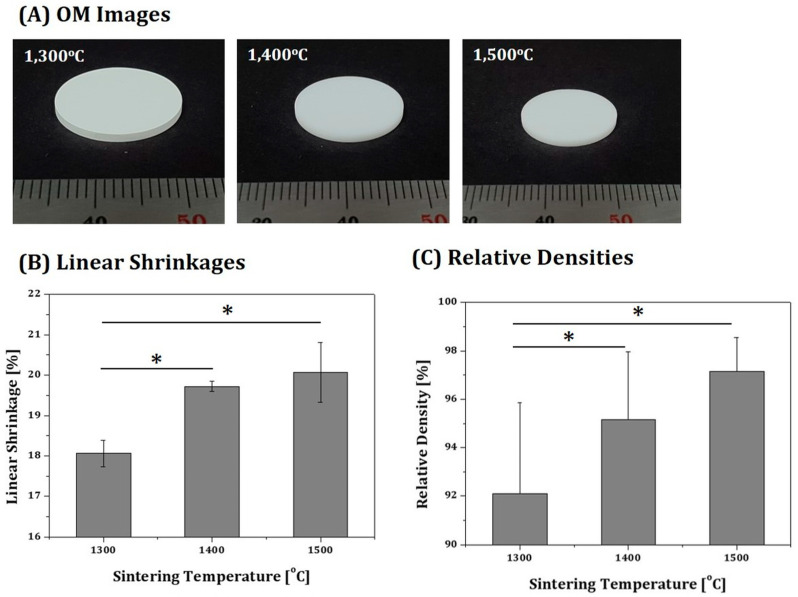
(**A**) Optical images of 4Y-PSZ disks sintered at various temperatures (1300 °C, 1400 °C, and 1500 °C) for 3 h (scale = 1 mm), and their corresponding (**B**) sintering shrinkages (* *p* < 0.05) and (**C**) relative densities (* *p* < 0.05).

After a multi-step debinding process using slow heating rates, 4Y-PSZ disks were sintered at various temperatures (1300 °C, 1400 °C, and 1500 °C) for 3 h, in order to evaluate their densification behaviors. Regardless of sintering temperatures, notable defects were not observed for any disks, as shown in Figure 9A. However, a higher sintering temperature resulted in a larger shrinkage (Figure 9B). Shrinkages of 18.06 ± 0.33%, 19.72 ± 0.13%, and 20.07 ± 0.74% were observed for the disks sintered at 1300 °C, 1400 °C, and 1500 °C, respectively. However, it should be noted that differences in sintering shrinkages of 4Y-PSZ components depending on locations are not significant, thus high dimensional accuracy can be obtained by carefully designing initial dimensions of 4Y-PSZ components. In addition, relative densities of sintered 4Y-PSZ disks increased with an increase in sintering temperature (Figure 9C). Relative densities of 92.10 ± 3.77%, 95.16 ± 2.79%, and 97.16 ± 1.38% were observed for the disks sintered at 1300 °C, 1400 °C, and 1500 °C, respectively.

### 3.8. Microstructures of Sintered 4Y-PSZ Disks

**Figure 10:**
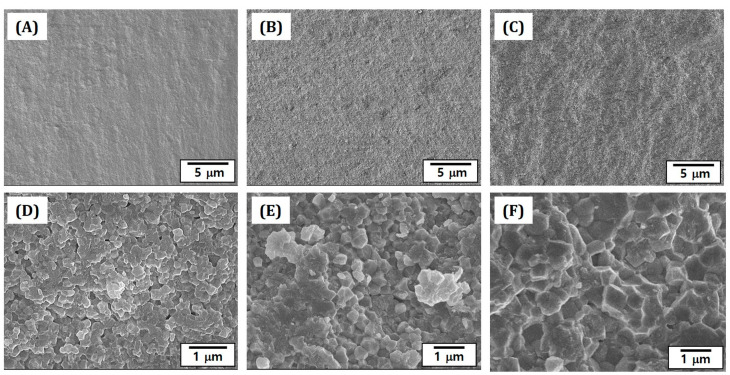
Representative FE-SEM images showing the fracture surfaces of 4Y-PSZ disks sintered for 3 h at various temperatures: 1300 °C (**A**,**D**), 1400 °C (**B**,**E**), and 1500 °C (**C**,**F**).

Microstructures of 4Y-PSZ disks sintered at various temperatures (1300 °C, 1400 °C, and 1500 °C) were closely characterized by FE-SEM. Their representative FE-SEM images were taken from fracture surfaces, which were obtained after biaxial flexural strength tests, as shown in Figure 10A–F. Regardless of sintering temperatures, all 4Y-PSZ disks showed no notable defects or visible interfaces between layers (Figure 10A–C). However, they showed different microstructures (Figure 10D–F), which were attributed to different densification behaviors and relative densities (c.f. Figure 9C). More specifically, when they were sintered at a relatively low temperature (1300 °C), a number of pores were observed due to the lack of densification between 4Y-PSZ particles (Figure 10D). However, pores were significantly reduced with an increase in sintering temperature up to 1400 °C (Figure 10E). In addition, a higher sintering temperature of 1500 °C allowed for high densification with only small residual pores at junctions between grains (Figure 10F). This finding suggests that 4Y-PSZ components manufactured by our photopolymerization-based 3D printing using a highly concentrated 4Y-PSZ suspension (solid loading = 48 vol%) with a camphor–HDDA vehicle could be highly densified at 1500 °C for 3 h, which could offer high mechanical properties and optical translucency for dental applications.

**Figure 11:**
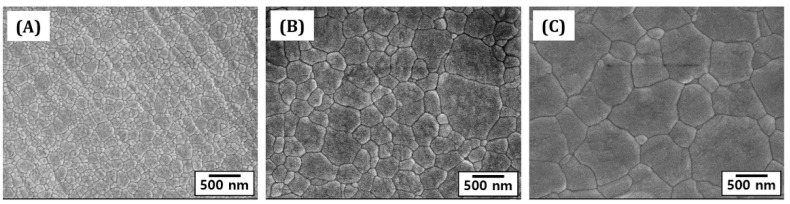
Representative FE-SEM images showing the free surfaces of 4Y-PSZ disks sintered for 3 h at various temperatures: (**A**) 1300 °C, (**B**) 1400 °C, and (**C**) 1500 °C.

The effect of sintering temperatures on the grain growth of 4Y-PSZ disks was examined by observing their free surfaces using FE-SEM. Results are shown in Figure 11A–C. Regardless of sintering temperature, relatively dense microstructures were observed for all 4Y-PSZ samples, different from their fracture surfaces. This is often the case with sintering behavior of ceramics [44]. On the other hand, grain growth became more pronounced at higher sintering temperatures.

Grain sizes measured by the linear intercept method increased notably with an increase in sintering temperature, as summarized in Table 3. Measured values for 4Y-PSZ samples sintered at 1300 °C, 1400 °C, and 1500 °C were 130 ± 8.9 nm, 275 ± 3.32 nm, and 543 ± 94.4 nm, respectively. Such increase in grain size was attributed to a great increase in driving force for grain growth at higher sintering temperatures. In addition, a longer dwelling time at given temperatures would increase grain size of 4Y-PSZ components, which would provide higher translucency but lower mechanical properties [6]. Thus, temperature and dwelling time for sintering should be controlled to tailor grain size of 4Y-PSZ components, while ensuring high densification, in order to provide desired mechanical and optical properties for dental applications.

### 3.9. Crystalline Phases of Sintered 4Y-PSZ 

**Figure 12:**
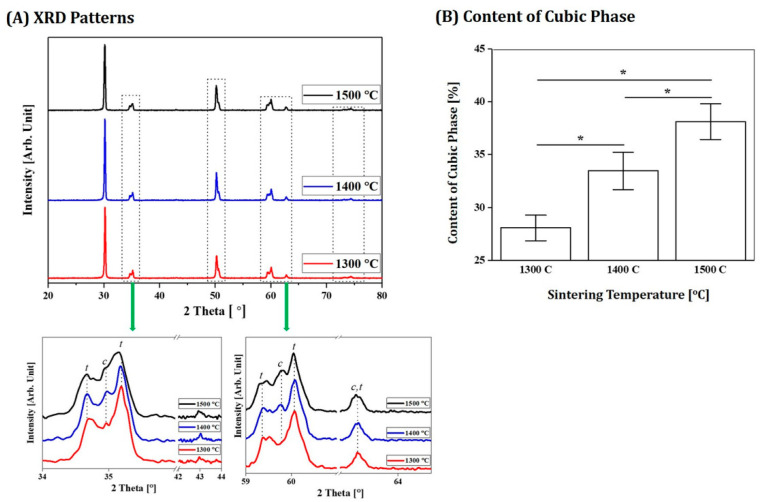
(**A**) Representative XRD patterns of 4Y-PSZ components sintered at various temperatures (1300 °C, 1400 °C, and 1500 °C) and (**B**) contents of cubic phase in components (* *p* < 0.05). “*t*” and “*c*” indicate tetragonal and cubic phases, respectively.

The 4Y-PSZ ceramics comprise two crystalline (tetragonal and cubic) phases. Their mechanical and optical properties are strongly affected by the contents of these two phases [1]. More specifically, higher cubic phase content can generally offer higher translucency [6,45]. Thus, the effect of sintering temperature on crystalline phases of 4Y-PSZ manufactured using our DLP technique was examined by XRD. Results are shown in Figure 12A. Regardless of sintering temperatures, all samples showed similar XRD patterns. More specifically, strong peaks associated with tetragonal (JCPDS card no. 01-079-1767) and cubic (JCPDS card no. 01-081-1550) phases were observed, while their relative intensities changed with different sintering temperatures. Thus, the contents of tetragonal and cubic phases were calculated by Rietveld refinement using X’Pert Highscore Plus software. As the sintering temperature increased, the content of the cubic phase increased, as shown in Figure 12B. The measured cubic phase content (~38.1%) after sintering at the highest temperature of 1500 °C is similar to those reported in the literature [6,27]. This finding suggests that 4Y-SZ components manufactured in this study could offer reasonable translucency when they are highly densified without notable defects.

### 3.10. Mechanical Properties of Sintered 4Y-PSZ 

**Figure 13:**
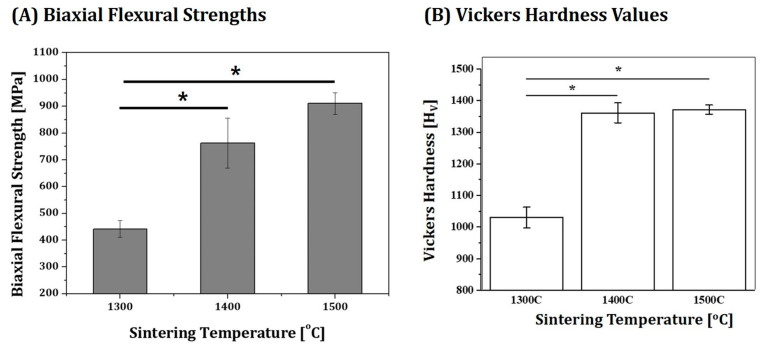
(**A**) Measured biaxial flexural strengths (* *p* < 0.05) and (**B**) Vickers hardness values of 4Y-PSZ disks sintered at various temperatures (1300 °C, 1400 °C, and 1500 °C) (* *p* < 0.05).

For uses as fixed prostheses in dentistry, 4Y-PSZ ceramics should satisfy required mechanical properties depending on their applications as described in ISO 6872 [34]. In other words, they should have flexural strengths higher than 300 MPa, 500 MPa, and 800 MPa for use as the single-unit, three-unit, and four-unit or more prostheses, respectively. Thus, flexural strengths of 4Y-PSZ disks sintered at various temperatures (1300 °C, 1400 °C, and 1500 °C) were measured by biaxial flexural strength tests. Measured values are summarized in Figure 13A. As the sintering temperature increased, the biaxial strength increased remarkably due to an increase in relative density (c.f. Figure 9C). It should be noted that 4Y-PSZ sintered at the highest temperature of 1500 °C for 3 h showed a reasonably high flexural strength (911 ± 40.7 MPa). In addition, Vickers hardness values increased from 1029 ± 32.8 H_v_ to 1371 ± 14.4 H_v_ with an increase in sintering temperature from 1300 °C to 1500 °C (Figure 13B). This increase was attributed to an increase in relative density.

Table 4 summarizes a brief review on flexural strengths and Vickers hardness values of zirconia components manufactured by DLP. Our values obtained after sintering at the highest temperature of 1500 °C were comparable to and even higher than those reported in the literature. Such high mechanical properties were attributed to achievement of high densification without notable defects. In addition, unlike traditional approaches, camphor used as the diluent in HDDA could recrystallize after the photocuring process, and camphor crystals could be removed favorably prior to removal of HDDA during the debinding process, thus minimizing the formation of defects.

It should be noted that temperature and dwelling time for sintering should be carefully controlled to tailor microstructures and crystalline structures of 4Y-PSZ components, in order to offer desired mechanical and optical properties. More specifically, sintering temperature should be sufficiently high (e.g., 1500 °C) to achieve almost full densification with high relative densities and should not be too high to avoid extensive grain growth with large grain sizes. In addition, a longer dwelling time for sintering can result in an increase in grain size. On the other hand, in terms of crystalline structure, it is important to tailor contents of tetragonal and cubic phases of 4Y-PSZ components. More specifically, yttria-doped zirconia ceramics can offer high mechanical properties since grains with tetragonal phase can transforme into those of monoclinic phase when stressed, thus hindering crack propagation [6]. Thus, as cubic phase content in 4Y-PSZ components increases, for example, by increasing sintering temperature in this study, translucency can increase, while mechanical properties will decrease. Thus, a sintering temperature of 1500 °C and a dwelling time of 3 h were employed in the present study to provide high mechanical properties (flexural strengths and hardness values).

### 3.11. Optical Properties of 4Y-PSZ

**Figure 14:**
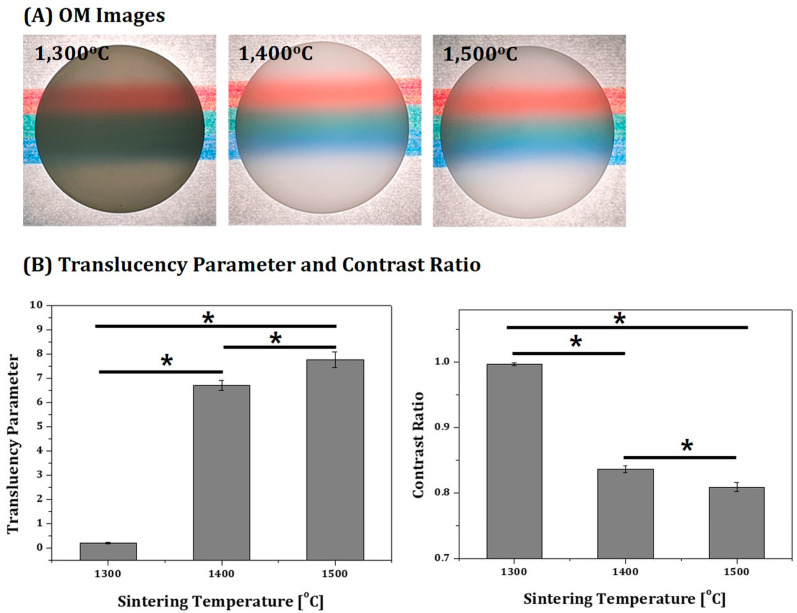
(**A**) Optical image of 4Y-PSZ disks sintered at various temperatures (1300 °C, 1400 °C, and 1500 °C) placed on colored lines and (**B**) their translucency (TP) and contrast ratio (CR) values (* *p* < 0.05).

In order to make full use of 4Y-PSZ components manufactured in this study for dental applications, it is very important to evaluate their optical properties (e.g., translucency and shade), since higher translucency is preferred in modern dentistry [1,4,6]. Thus, optical properties of 4Y-PSZ disks sintered at various temperatures (1300 °C, 1400 °C, and 1500 °C) were roughly examined by observing their light transmittance capability. Disks with a thickness of 1 mm were placed onto colored lines, as shown in Figure 14A. As the sintering temperature increased, colored marks became more visible through disks. This improved light transmittance capability was mainly attributed to a reduction in porosity, since pores would scatter incident light [48,49].

To more closely evaluate the optical properties of 4Y-PSZ disks sintered at various temperatures (1300 °C, 1400 °C, and 1500 °C), their translucency parameter (TP) and contrast ratio (CR) were measured by CIE colorimetry. The TP and CR values computed from Equations (5) and (6), respectively, are displayed in Figure 14B. When sintered at the lowest temperature of 1300 °C, the 4Y-PSZ disk showed very low TP and very high CR values, indicating very low translucency due to a low relative density. On the other hand, as the sintering temperature increased, the TP value increased, whereas the CP value decreased due to an increase in densification with a reduction in porosity. However, even after sintering at the highest temperature of 1500 °C, 4Y-PSZ disks had lower translucency (TP = 7.77 ± 0.32 and CR = 0.809 ± 0.007) than commercially available 4Y-PSZ blocks [50,51,52] used for dental computer-aided design and computer-aided manufacturing (CAD/CAM) technology. This reduction in translucency was mainly attributed to the presence of residual pores and defects after sintering, which is often the case with zirconia ceramics manufactured photopolymerization-based 3D printing [27,53]. However, translucency of zirconia ceramics would be enhanced by more uniformly dispersing zirconia particles with higher contents in photopolymerizable vehicles and more tightly controlling parameters for layer-by-layer 3D printing process.

### 3.12. Usefulness of Present Approach for Dental Applications

**Figure 15:**
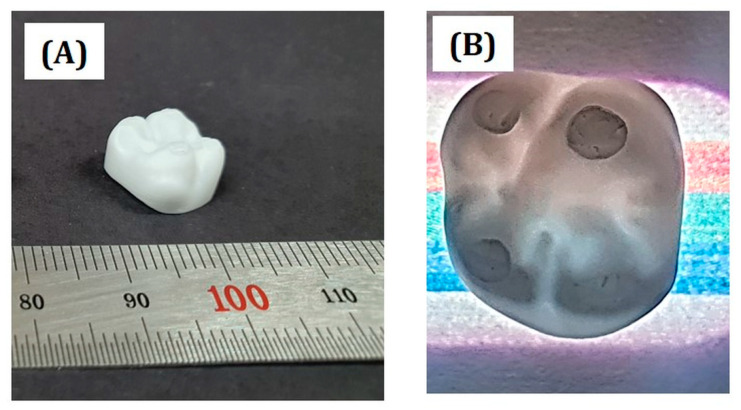
Optical images showing (**A**) 3D shape of a 4Y-PSZ dental crown sintered at 1500 °C for 3 h and (**B**) its translucency placed onto colored lines under light illumination.

The main purpose of the present study was to demonstrate the utility of zirconia suspensions prepared using an HDDA/camphor vehicle for photopolymerization-based 3D printing, which would be utilized to manufacture 4Y-PSZ components for dental applications, including dental crowns, abutments, and implants. Thus, dental crowns made of 4Y-PSZ were manufactured using our approach. After sintering at 1500 °C for 3 h, no notable defects, such as severe distortion or cracks, were observed even for a complicated shape, as shown in Figure 15A. In addition, dental crowns were translucent under light illumination (Figure 15B). Although more detailed studies should be carried out to examine the accuracy of our photopolymerization-based 3D printing, it is reasonable to suppose that our approach could be successfully used to manufacture a variety of 4Y-PSZ components for dental applications.

## 4. Conclusions

The use of a 65 wt% HDDA/35 wt% camphor solution as a photopolymerizable vehicle enabled highly concentrated 4Y-PSZ suspensions (solid loading = 48 vol%) to have reasonably low viscosities (1399 ± 55.8 mPa·s at a shear rate of 75 s^−1^). Thus, 4Y-PSZ components could be successfully manufactured by our photopolymerization-based 3D printing. To obtain desired mechanical and optical properties for dental applications, several processing parameters were tightly controlled, including UV illumination time to completely photopolymerize 50-μm-thick layers of 4Y-PSZ suspensions with good bonding between deposited layers, designing of initial dimensions of 4Y-PSZ components to offer high accuracy, a debinding process to remove photopolymerized HDDA and organic phases without defects, and sintering temperature (1300–1500 °C) to obtain high densification. The 4Y-PSZ components sintered at the highest temperature of 1500 °C and high relative density of ~97.2% could be obtained. Thus, high mechanical properties (biaxial flexural strength = ~911 MPa and Vickers hardness = 1371 ± 14.4 H_v_) and a reasonable optical translucency (TP = ~7.77 and CR = ~0.809) could be obtained, which would be useful for dental applications.

## Figures and Tables

**Table 1:** Constituents of zirconia suspensions prepared using an HDDA–camphor solution as a liquid vehicle for DLP process.

Role	Material (Supplier)	Weight [g]
Zirconia Powder	Yttria-stabilized zirconia (Zpex4) (Tosoh Co., Tokyo, Japan)	71.85
Photocurable Monomer	1,6-hexanediol diacrylate (HDDA) (Sartomer, PA, USA)	6.5
Diluent	Camphor (C_10_H_16_O) (Sigma Aldrich, St. Louis, MO, USA)	3.5
Dispersant	Solution of a structured acrylate copolymer with pigment-affinic groups (DISPERBYK-2001) (BYK-Chemie GmbH, Wesel, Germany)	2.87
Photo Initiator	Diphenyl(2,4,6-trimethylbenzoyl) phosphine oxide (TPO) (Sigma Aldrich, St. Louis, MO, USA)	1.13

**Table 2:** A brief review on viscosities of zirconia suspensions used for DLP process.

Refs.	[21]	[23]	[24]	[27]	Present Study
Solid Loading	75 wt%	42 vol%	44 vol%	50 vol%	48 vol%
Viscosities [Pa·s]	1.6	4.88	~2	~3.5	~1.40

**Table 3:** Grain sizes of 4Y-PSZ disks sintered at various temperatures (1300 °C, 1400 °C, and 1500 °C).

Sintering Temperatures [°C]	1300	1400	1500
Grain Size [nm]	130 ± 8.9	275 ± 32.3	543 ± 94.4

**Table 4:** A brief review on mechanical properties of zirconia components manufactured by DLP process.

**Refs.**	**[10]**	**[24]**	**[25]**	**[26]**	**[27]**
Flexural strength [MPa]	1042	741	539	539.1	831
Hardness	12.59 GPa	n/a	13.02 GPa	1556 H_v_	n/a
**Refs.**	**[28]**	**[29]**	**[46]**	**[47]**	**Present Study**
Flexural strength [MPa]	n/a	530.25	433	n/a	911
Hardness	13.0597 H_v_	17.76 GPa	1848 H_v_	12.59 GPa	1371 H_v_

## Data Availability

Not applicable.

## References

[1] Manicone P.F., Iommetti P.R., Raffaelli L. (2007). An overview of zirconia ceramics: Basic properties and clinical applications. J. Dent..

[2] Denry I., Kell J. (2008). State of the art of zirconia for dental applications. Dent. Mater..

[3] Miyazaki T., Nakamura T., Matsumura H., Ban S.J., Kobayashi T. (2013). Current status of zirconia restoration. J. Prosthodont. Res..

[4] Zhang Y., Lawn B.R. (2018). Novel zirconia materials in dentistry. J. Dent. Res..

[5] Mao M.R., Kaizer M.R., Zhao M., Guo B., Song Y.F., Zhang Y. (2018). Graded ultra-translucent zirconia (5Y-PSZ) for strength and functionalities. J. Dent. Res..

[6] Zhang F., Reveron H., Spies B.C., Van Meerbeek B., Chevalier J. (2019). Trade-off between fracture resistance and translucency of zirconia and lithium-disilicate glass ceramics for monolithic restorations. Acta Biomater..

[7] Fonseca Y.R., Elia C.N., Monteiro S.N., Santos H.E.S.D., Santos C.D. (2019). Modeling of the influence of chemical composition, sintering temperature, density, and thickness in the light transmittance of four zirconia dental prostheses. Materials.

[8] Galante R., Figueiredo-Pina C.G., Serro A.P. (2019). Additive manufacturing of ceramics for dental applications: A review. Dent. Mater..

[9] Khanlar L.N., Rios A.S., Tahmaseb A., Zandinejad A. (2021). Additive manufacturing of zirconia ceramic and its application in clinical dentistry: A review. Dent. J..

[10] Sun J., Chen X., Wade-Zhu J., Binner J., Bai J. (2021). A comprehensive study of dense zirconia components fabricated by additive manufacturing. Addit. Manuf..

[11] Zakeri S., Vippola M., Levänen E. (2020). A comprehensive review of the photopolymerization of ceramic resins used in stereolithography. Addit. Manuf..

[12] de Camargo I.L., Morais M.N., Fortulan C.A., Branciforti M.C. (2021). A review on the rheological behavior and formulations of ceramic suspensions for vat photopolymerization. Ceram. Inter..

[13] Harianawala H.H., Kheur M.G., Apte S.K., Kale B.B., Sethi T.S., Kheur S.M. (2014). Comparative analysis of transmittance for different types of commercially available zirconia and lithium disilicate materials. J Adv. Prosthodont..

[14] Manziuc M.M., Gasparik C., Negucioiu M., Constantiniuc M., Burde A., Vlas I., Dudea D. (2019). Optical properties of translucent zirconia: A review of the literature. EBT J..

[15] Harrer W., Schwentenwein M., Lube T., Danzer R. (2017). Fractography of zirconia-specimens made using additive manufacturing (LCM) technology. J. Eur. Ceram. Soc..

[16] Jang K.J., Kang J.H., Sakthiabirami K., Lim H.P., Yun K.D., Yim E.K., Oh G.J., Yang H.S., Lee K.K., Park S.W. (2019). Evaluation of cure depth and geometrical overgrowth depending on zirconia volume fraction using digital light processing. J. Nanosci. Nanotechno..

[17] Jang K.J., Kang J.H., Fisher J.G., Park S.W. (2019). Effect of the volume fraction of zirconia suspensions on the microstructure and physical properties of products produced by additive manufacturing. Dent. Mater..

[18] Wang L., Liu X., Wang G., Tang W., Li S., Duan W., Dou R. (2020). Partially stabilized zirconia moulds fabricated by stereolithographic additive manufacturing via digital light processing. Mater. Sci. Eng. A.

[19] Shen M., Zhao W., Xing B., Sing Y., Gao S., Wang C., Zhao Z. (2020). Effects of exposure time and printing angle on the curing characteristics and flexural strength of ceramic samples fabricated via digital light processing. Ceram. Int..

[20] Mei Z., Lu Y., Lou Y., Yu P., Sun M., Tan X., Zhang J., Yue L., Yu H. (2021). Determination of hardness and fracture toughness of Y-TZP manufactured by digital light processing through the indentation technique. BioMed Res. Int..

[21] Komissarenko D.A., Sokolov P.S., Evstigneeva A.D., Shmeleva I.A., Dosovitsky A.E. (2018). Rheological and curing behavior of acrylate-based suspensions for the DLP 3D printing of complex zirconia parts. Materials.

[22] Santoliquido O., Colombo P., Ortona A. (2019). Additive manufacturing of ceramic components by digital light processing: A comparison between the “bottom-up” and the “top-down” approaches. J. Eur. Ceram. Soc..

[23] Li X., Zhong H., Zhang J., Duan Y., Bai H., Li J., Jiang D. (2020). Dispersion and properties of zirconia suspensions for stereolithography. Int. J. Appl. Ceram. Technol..

[24] Borlaf M., Serra-Capdevila A., Colominas C., Graule T. (2019). Development of UV-curable ZrO_2_ slurries for additive manufacturing (LCMDLP) technology. J. Eur. Ceram. Soc..

[25] Jiang C.P., Hsu H.J., Lee S.Y. (2014). Development of mask-less projection slurry stereolithography for the fabrication of zirconia dental coping. Int. J. Precis. Eng. Manuf..

[26] Lee S.Y., Jiang C.P. (2015). Printing system using dynamic mask projection for fabricating zirconia dental implants. Mater. Manuf. Process..

[27] Kim J.H., Maeng W.Y., Koh Y.H., Kim H.E. (2020). Digital light processing of zirconia prostheses with high strength and translucency for dental applications. Ceram. Int..

[28] He R., Liu W., Wu Z., An D., Huang M., Wu H., Jiang Q., Ji X., Wu S., Xie Z. (2018). Fabrication of complex-shaped zirconia ceramic parts via a DLP-stereolithography-based 3D printing method. Ceram. Inter..

[29] Wu H., Liua W., He R., Wu Z., Jiang Q., Song X., Chen Y., Cheng L., Wu S. (2017). Fabrication of dense zirconia-toughened alumina ceramics through a stereolithography-based additive manufacturing. Ceram. Int..

[30] Lee Y.H., Lee J.B., Maeng W.Y., Koh Y.H., Kim H.E. (2019). Photocurable ceramic slurry using solid camphor as novel diluent for conventional digital light processing (DLP) process. J. Eur. Ceram. Soc..

[31] Lee Y.H., Lee J.W., Yang S.Y., Lee H., Koh Y.H., Kim H.E. (2021). Dual-scale porous biphasic calcium phosphate gyroid scaffolds using ceramic suspensions containing polymer microsphere porogen for digital light processing. Ceram. Inter..

[32] Tomeckova V., Halloran J.W. (2012). Porous ceramics by photopolymerization with terpene–acrylate vehicles. J. Am. Ceram. Soc..

[33] (2021). Standard Test Methods for Determining Average Grain Size.

[34] (2008). Dentistry-Ceramic Materials.

[35] Miura D., Ishida Y., Miyasaka T., Aoki H., Shinya A. (2020). Reliability of different bending test methods for dental press ceramics. Materials.

[36] (2015). Standard Test Method for Vickers Indentation Hardness of Advanced Ceramics.

[37] Johnston W.M. (2014). Review of translucency determinations and applications to dental materials. J. Esthet. Restor. Dent..

[38] Miyagawa Y., Powers J.M., O’Brien W.J. (1981). Optical properties of direct restorative materials. J. Dent. Res..

[39] Ragain J.C. (2016). A review of color science in dentistry: Colorimetry and color space. J. Dent. Oral. Disord. Ther..

[40] Vichi A., Sedda M., Fonzar R.F., Carrabba M., Ferrari M. (2016). Comparison of contrast ratio, translucency parameter, and flexural strength of traditional and “augmented translucency” zirconia for CEREC CAD/CAM System. J. Esthet. Restor. Dent..

[41] Pan Q., Westland S. (2018). Tooth color and whitening—Digital technologies. J. Dent..

[42] Griffith M.L., Halloran J.W. (1997). Scattering of ultraviolet radiation in turbid suspensions. J. Appl. Phys..

[43] Halloran J.W. (2016). Ceramic stereolithography: Additive manufacturing for ceramics by photopolymerization. Annu. Rev. Mater. Res..

[44] Bordia R.K., Kang S.J.L., Olevsky E.A. (2017). Current understanding and future research directions at the onset of the next century of sintering science and technology. J. Am. Ceram. Soc..

[45] Lim C.H., Vardhaman S., Reddy N., Zhang Y. (2022). Composition, processing, and properties of biphasic zirconia bioceramics: Relationship to competing strength and optical properties. Ceram. Inter..

[46] Jang J.G., Kang J.-H., Joe K.-B., Sakthiabirami K., Jang K.-J., Jun M.-J., Oh G.-J., Park C., Park S.-W. (2022). Evaluation of Physical Properties of Zirconia Suspension with Added Silane Coupling Agent for Additive Manufacturing Processes. Materials.

[47] Sarwar W.A., Kang J.-H., Yoon H.-I. (2021). Optimized Zirconia 3D Printing Using Digital Light Processing with Continuous Film Supply and Recyclable Slurry System. Materials.

[48] Lee Y.K. (2016). Criteria for clinical translucency evaluation of direct esthetic restorative materials. Restor. Dent. Endod..

[49] Shahmiri R., Standard O.C., Hart J.N., Sorrell C.C. (2018). Optical properties of zirconia ceramics for esthetic dental restorations: A systematic review. J. Prosthet. Dent..

[50] Sulaiman T.A., Abdulmajeed A.A., Donovan T.E., Ritter A.V., Vallittu P.K., Narhi T.O., Lassila L.V. (2015). Optical properties and light irradiance of monolithic zirconia at variable thicknesses. Dent. Mater..

[51] Sen N., Isler S. (2020). Microstructural, physical, and optical characterization of high-translucency zirconia ceramics. J. Prosthet. Dent..

[52] Pizzolatto G., Borba M. (2021). Optical properties of new zirconia-based dental ceramics: Literature review. Ceramics.

[53] Xiang D., Xu Y., Bai W., Lin H. (2021). Dental zirconia fabricated by stereolithography: Accuracy, translucency and mechanical properties in different build orientations. Ceram. Inter..

